# Phenotypes of Non-Attached *Pseudomonas aeruginosa* Aggregates Resemble Surface Attached Biofilm

**DOI:** 10.1371/journal.pone.0027943

**Published:** 2011-11-21

**Authors:** Morten Alhede, Kasper Nørskov Kragh, Klaus Qvortrup, Marie Allesen-Holm, Maria van Gennip, Louise D. Christensen, Peter Østrup Jensen, Anne K. Nielsen, Matt Parsek, Dan Wozniak, Søren Molin, Tim Tolker-Nielsen, Niels Høiby, Michael Givskov, Thomas Bjarnsholt

**Affiliations:** 1 Institute for International Health, Immunology and Microbiology, University of Copenhagen, Copenhagen, Denmark; 2 Department of Biomedical Sciences, University of Copenhagen, Copenhagen, Denmark; 3 Department of Microbiology, University of Washington, Seattle, Washington, United States of America; 4 Department of Clinical Microbiology, Rigshospitalet, Copenhagen, Denmark; 5 Division Infectious Disease, Center for Microbial Interface Biology, The Ohio State University, Columbus, Ohio, United States of America; 6 Department of Systems Biology, Technical University of Denmark, Lyngby, Denmark; University of Birmingham, United Kingdom

## Abstract

For a chronic infection to be established, bacteria must be able to cope with hostile conditions such as low iron levels, oxidative stress, and clearance by the host defense, as well as antibiotic treatment. It is generally accepted that biofilm formation facilitates tolerance to these adverse conditions. However, microscopic investigations of samples isolated from sites of chronic infections seem to suggest that some bacteria do not need to be attached to surfaces in order to establish chronic infections. In this study we employed scanning electron microscopy, confocal laser scanning microscopy, RT-PCR as well as traditional culturing techniques to study the properties of *Pseudomonas aeruginosa* aggregates. We found that non-attached aggregates from stationary-phase cultures have comparable growth rates to surface attached biofilms. The growth rate estimations indicated that, independently of age, both aggregates and flow-cell biofilm had the same slow growth rate as a stationary phase shaking cultures. Internal structures of the aggregates matrix components and their capacity to survive otherwise lethal treatments with antibiotics (referred to as tolerance) and resistance to phagocytes were also found to be strikingly similar to flow-cell biofilms. Our data indicate that the tolerance of both biofilms and non-attached aggregates towards antibiotics is reversible by physical disruption. We provide evidence that the antibiotic tolerance is likely to be dependent on both the physiological states of the aggregates and particular matrix components. Bacterial surface-attachment and subsequent biofilm formation are considered hallmarks of the capacity of microbes to cause persistent infections. We have observed non-attached aggregates in the lungs of cystic fibrosis patients; otitis media; soft tissue fillers and non-healing wounds, and we propose that aggregated cells exhibit enhanced survival in the hostile host environment, compared with non-aggregated bacterial populations.

## Introduction

Many bacterial species are capable of adopting either a planktonic or sessile state. Planktonic bacteria are classically defined as single cells in suspension whereas sessile, aggregated cells are defined by the American Center for Disease Control (CDC) as “an assemblage of microbial cells that is irreversibly associated (not removed by gentle rinsing) with a surface and enclosed in a matrix of primarily polysaccharide material” [1]. Most researchers refer to this as the biofilm mode of growth. Much of the recent literature generally agrees that bacteria growing as biofilms (attached to a surface), are the major cause of chronic infections [2]. In fact, the presence of biofilms in an infection is thought to equate to a chronic infection [3], [4], whereas planktonic pathogens are generally associated with acute infections. This clinical impact of biofilms has inspired researchers to investigate the biofilm mode of growth intensively in the recent years.

Biofilm formation represents a bacterial survival strategy in unfriendly environments. A wide range of harsh conditions challenges the bacteria as they invade their hosts. For establishing a successful infection, bacteria must cope with low levels of iron, oxidative stress, macerating enzymes, phagocytic cells and other host defense mechanisms, and those antimicrobials administered by the physicians. Much evidence supports the view that biofilms can survive the antimicrobial treatment during infections [5], [6], [7], [8], [9]. This complex protection relies to a certain extend on general resistance mechanisms including efflux pumps and enzymatic modifications in addition to tolerance derived from biofilm structure [10]. The biofilm matrix reduces exposure of the resident cells to host immune cells and antibiotics [9], [11]. Bacterial cells buried within biofilms have limited access to nutrients, which reduces metabolic activity and subsequently limits the effectiveness of most antibiotics [12].

The shielding effect of the biofilm matrix-encased bacteria has several beneficial consequences during host encounters. The decreased penetration of phagocytes is an obvious example. However, the activation of phagocytes (respiratory burst) and the complement system, have also been shown to be markedly impaired against biofilm populations [13], [14], [15]. Interestingly, this impairment of host immunity is lost upon mechanical disruption of the biofilm into individual cells [16], [17].

Recent *ex vivo* data has yielded valuable insights in the spatial distribution of bacteria in chronic wound infections, lungs and soft tissue fillers etc. [18], [19], [20], [21]. Peptide nucleic acid (PNA) fluorescence *in situ* hybridization (FISH) and confocal laser scanning microscopy (CLSM) imaging of samples isolated from sites of chronic human infections demonstrate that bacteria do not inevitably attach to surfaces. Rather, they attach to their fellow bacteria likely by means of matrix components and mucus, and they seem to establish impenetrable barriers to the host e.g. phagocytic cells [19], [22]. Thus, it appears that opposed to harboring bacteria firmly attached to a surface, chronic infections can be characterized by aggregates suspended within host tissue or lumens of organs.

Aggregating bacteria have been discussed for years and are very important in environmental microbiology [23], however most attention within medical research has been given to the study of biofilm bacteria that are clearly surface attached. Therefore we investigated if non-attached, bacterial microcolonies (referred to as aggregates), similar to those observed from patient samples, exhibit many of the medical relevant characteristics of surface attached biofilms. This distinction surface-attached aggregates vs. suspended aggregates and whether they both represent the biofilm life style has been a point of controversy in recent years. In the present study we have compared aggregates of *P. aeruginosa* with surface attached biofilms. Our experiments indicate that non-surface attached aggregates of bacteria represent the biofilm mode of growth.

## Results

### SEM investigations of aggregates and surface attached biofilms

**Figure 1:**
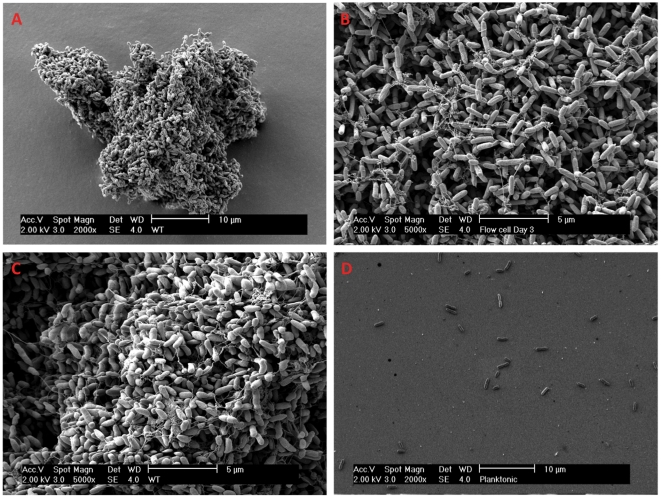
SEM survey of different *P. aeruginosa* cultures. SEM of A – Aggregate harvested from a 48-h old stationary culture. B – Details of a 3-day old biofilm grown in flow-cell. C – Details of 48-h old stationary aggregate. D – Planktonic cells (OD_600_ = 0.5). Note that the single planktonic cells are difficult to fixate on the specimen during SEM preparation due to the small size (leads to few cells on the specimen).

The matrix of surface attached biofilms has been shown to consist of various interconnecting polymers such as polysaccharides, DNA, peptides etc. To investigate whether if non-attached aggregates carry similar interconnecting fibers and matrix polymers, we harvested macroscopic aggregates (Microcopic appearance seen in Figure 1A) from a 48-h static culture setting and compared them to both planktonic cells and a 3-days-old surface -attached biofilm.

Interestingly, we found that aggregates and surface attached biofilms showed a striking similarity with regards to visual structure. The cells inside the non-attached aggregates seemed to be interconnected by fiber-like structures that resembled those found in the surface attached biofilms (e.g. flow-cell biofilms) (Figure 1B+C). In the planktonic settings only single cells were observed (Figure 1D).

### Aggregates arise from non-clonal growth

**Figure 2:**
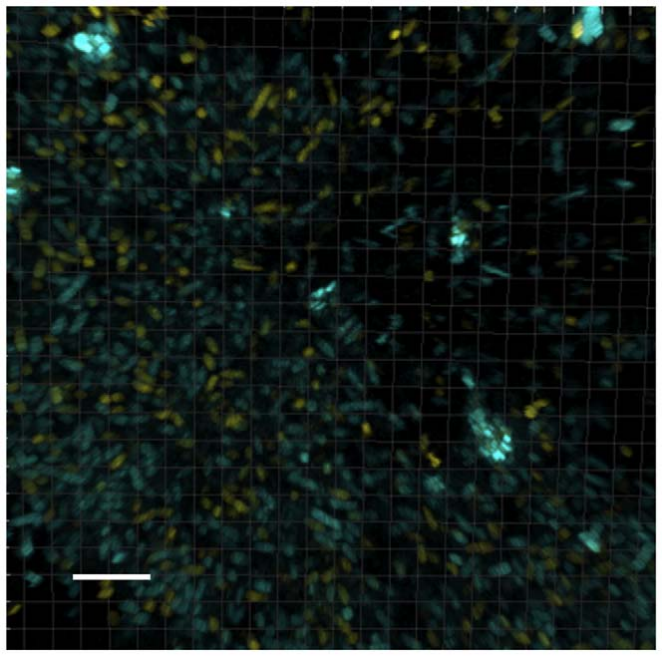
Invstigation of the clonal relationship in clumps. Mixed Yellow (YFP) and Cyan (CFP) constitually tagged PAO1 were inoculated together. The CLSM visualization demonstrated that the clumps consisted of both yellow and blue cells indicating non-clonal growth. Length of size bar: 6 µm.

The formation of the initial microcolonies (the stalks) in *P. aeruginosa* surface attached biofilms is due to clonal growth, however the mushroom caps of the biofilms are formed via non-clonal populations migrating to the top of the cap [24]. To investigate if aggregates were initiated by clonal growth, we inoculated both Yellow (YFP) and Cyan (CFP) tagged PAO1 in a tissue culture test plate (Figure 2). Using CLSM we observed that the formed aggregates consisted of both yellow and blue cells indicating that clonal growth is not the primary cause of aggregate formation. This indicates that formation of non-surface attached aggregates may proceed through the same pathway as the mushroom caps.

### Aggregates display heightened antibiotic tolerance

**Figure 3:**
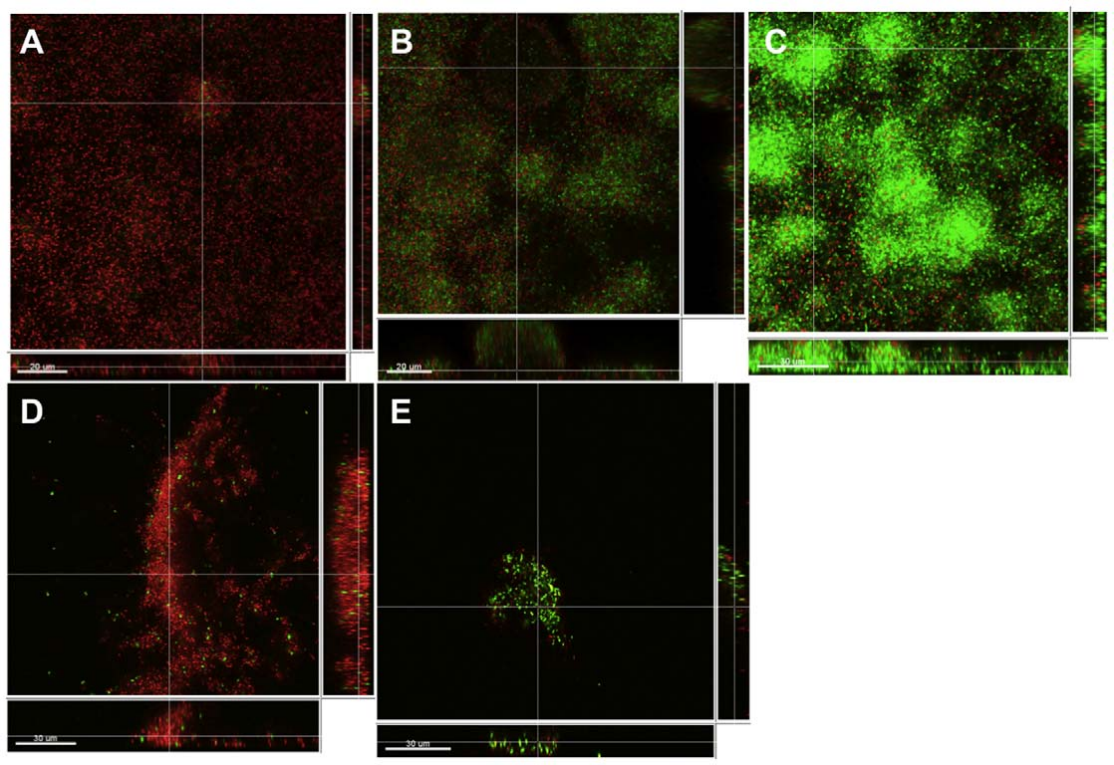
Antibiotic tolerance of maturing flow-cell biofilms of *P. aeruginosa* and aggregates harvested from a static *P. aeruginosa* culture. Biofilms and aggregates were grown for 24 h to 72-h prior to tobramycin (100 ug/ml) treatment for 24-h. For visualization by CLSM a GFP-tagged PAO1 strain (green) was used and stained with the DNA stain PI (red) for visualizing dead bacteria. Panels A, B and C represent biofilm tolerance on day 1, 2 and 3 respectively andpanels D and E are aggregates on day 1 and 2. Length of size bars: 20 µm.

Surface attached biofilms show an increased tolerance to antibiotic exposure compared with their planktonic counterparts [12], [25], [26]. We speculated whether aggregates also show this increased antibiotic tolerance. Therefore the aggregated cells were isolated and exposed to 100 µg/ml tobramycin for 24-h (which is 100 fold above the planktonic MIC values). To visualize the survival of the aggregating cells, propidium iodiode (PI) was added together with tobramycin. CLSM visualization indicated that the non-surface attached aggregates behaved similarly to surface attached biofilms with respect to increased tolerance to the antibiotic tobramycin (Figure 3). The tolerance for both aggregates and biofilms were found to increase with age. We found that both 1-day-old aggregates (Figure 3D) and 1-day-old biofilm (Figure 3A) were very susceptible to tobramycin. However, on day 2 the aggregates (Figure 3E) were found tolerant whereas a comparable tolerance was first seen on day 3 in the biofilm (Figure 3C). The difference in “age of tolerance” could be due to the continuous access to media in the case of the biofilm.

### Growth rates in aggregates and biofilms

We speculated that the limited effect of tobramycin on aggregate and biofilm cells could be due to a lower metabolic activity. In order to get an indication of the growth rate possessed by both aggregates and biofilm we decided to measure rRNA content by RT-PCR since the number of ribosomes fluctuates linearly with growth rate [27].

**Figure 4:**
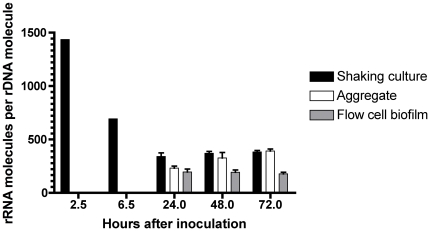
Growth rates of flow-cell biofilms and aggregates over time. Growth rates were estimated by quantifying rRNA molecules per rDNA molecules by RT-PCR.

As seen from Figure 4 we found that the number of rRNA molecules normalized to the number of rDNA molecules in both aggregate and biofilm cells are comparable to late stationary phase planktonic cells. Hence it seems that the limited effect of tobramycin can be explained by low growth. However, we see that the number rRNA molecules and thus the growth rate do not change significantly with age of the aggregate or biofilm. This is interesting since we found that the tolerance towards tobramycin increases with the age of the aggregates and biofilm. Therefore the tolerance must rely on several factors in addition to slow growth.

The limited effect of tobramycin activity on the aggregates could thus also be related to both a reduced penetration into the aggregate and the fact that tobramycin needs oxygen in order to enter metabolically active bacterial cells and bind to 30S ribosomes [28]. Such active cells are not present in the depth of microbial biofilms because of microaerobic/anaerobic environmental conditions [28], [29], explaining the selective biofilm activity of tobramycin and possibly also in our aggregates.

### Quantitative tolerance to tobramycin and colistin

**Figure 5:**
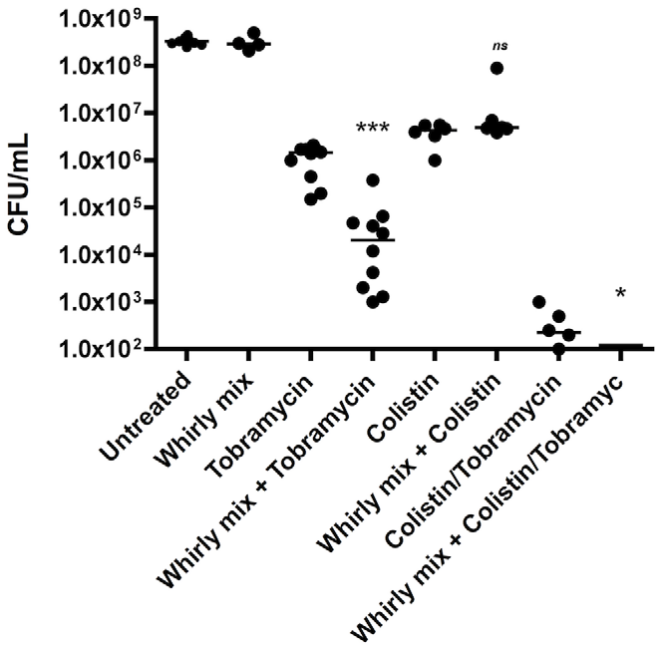
Antibiotic tolerance of 48-h old aggregates harvested from a static *P. aeruginosa* culture and the effect of disruption by whirly mixing. Aggregates were grown for 48-h prior to tobramycin (100 ug/ml) treatment for 24-h or whirly mix (20 seconds) followed by tobramycin treatment for 24 h. 24 h. Bars represent the median. Mann-Whitney test were chosen to compare the medians of the different treatment regimens. ns indicates no significant differences between treatments (p>0.05). * Indicates significant difference (p<0.05). *** Indicates a very significant difference (p<0.0001).

To obtain a quantitative measure for the antibiotic tolerance, we determined the number of viable bacteria after 24 h of antibiotic exposure. We used two types of antibiotics, which are known to kill either growing or non-growing bacteria (100 µg/ml Tobramycin and 25 µg/ml Colistin, respectively). Both treatment regimes were found inadequate in killing all bacteria within the aggregates (Figure 5). As a control we found that the same treatment could completely eliminate survival of planktonic growing bacteria (OD_600_ = 0.1) in the case of tobramycin and reduced the CFU with 6 log units in the case of colistin. Interestingly, the combination of the two treatments increased the killing of the aggregates synergistically. Similar to previous reports of surface attached biofilms [30], [31], we found that the non-attached aggregates consist of at least two subpopulations; one population showing tolerance to tobramycin treatment and one showing tolerance to colistin, but application of both drugs killed a significant larger fraction of the bacteria found in the non-surface attached aggregates (p<0.05, Mann-Whitney test).

With this established, we investigated whether if tolerance could be caused by the physiology assumed by cells within the aggregates or simply due to mutational changes. We therefore disrupted the aggregates by mechanical mixing. We found that physical disruption of the aggregates caused a significantly (p<0.05, Mann-Whitney test) lower tolerance to tobramycin treatment but not to colistin (Figure 5). This is interesting since bacteria (according to [30]), which exhibit high metabolic activity, are able to survive the colistin treatment, in contrast to subpopulations exhibiting low metabolic activity that are tolerant to tobramycin. It was suggested by [30] that an energy-driven adaptation response might be required to leave the cells in the upper part of a flow-cell biofilm tolerant to colistin. Thus, it seems that the bacteria are protected by the physiology within the aggregate and that we might induce metabolic activity by disrupting the aggregates. A similar effect was found by sonicating the aggregates in an ultrasonic bath (data not shown).

**Figure 6:**
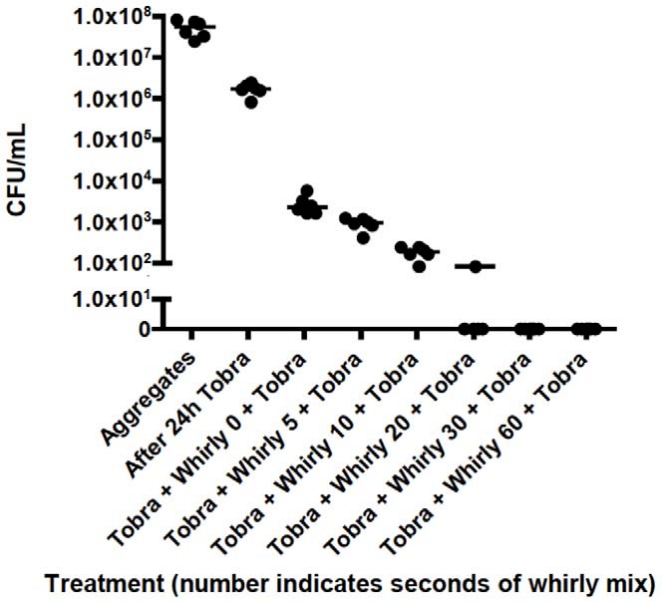
Effect of prolonged whirly mix. Aggregates were grown for 48-h prior to tobramycin (100 ug/ml) treatment for 24-h, followed by whirly mix and another round of tobramycin treatment. Number indicates seconds of whirly mix. Bars represent the median. Kruskal-Wallis with Dunn's post test was chosen to compare the effect of whirly mix and subsequent treatment with tobramycin. The post test found the increased killing to be significant (P<0.05 when compared to 0 seconds) after 30 seconds of whirly mix.

To test whether the increased tolerance of aggregates is reversible (e.g. caused by physiology in the aggregate), we treated the aggregates with tobramycin for 24-h and subsequently disrupted the aggregates (Figure 6) mechanically prior to a second, 24-h antibiotic treatment. The second round of antibiotic treatment killed all bacteria from the disrupted aggregates in a time dependent manner (Cuzick's test of trend p<0.0001), e.g. extended periods of mixing/biofilm-disruption increased the extent of antibiotic tobramycin mediated killing. We found the increased killing to be significant (Kruskal-Wallis with Dunn's post test: P<0.05 when compared to 0 seconds) after 30 seconds of whirly mix. These results indicate that the capacity of the aggregates to cope with exposure to tobramycin treatment is reversible and thus likely to be caused by the immediate physiological state and/or the presence of particular matrix components in the aggregates rather than being caused by mutations leading to resistance. This is not surprising, since penetration of tobramycin into *P. aeruginosa* colony biofilms has been shown to be impeded and that breaking up of the biofilm cells restored their antibiotic sensitivity [28]. This emphasizes the apparent similarities between surface attached biofilms and aggregates. On the other hand, mechanical disruption of the aggregates did not increase susceptibility to colistin treatment (Figure 5).

The fact that the biological state of aggregated bacteria is important for surviving environmental stresses is supported by the study of Barclay *et al.* (1996). They showed that *P. aeruginosa* cells isolated from the sputum of CF patients 4-h after intravenous administration of tobramycin, displayed increased tolerance to subsequent *in vitro* tobramycin treatment, but this increased tolerance was lost during the following 24 to 48-h. This indicates that the observed tolerance was due to physiological adaptations and not mutations [32].

### Resistance to phagocytes

Surface attached biofilms have been shown to impair the oxidative burst of neutrophils [16], [17], [33]. We have previously found that impairment of PMNs is due to the presence of rhamnolipid produced by surface attached biofilms [33], which functions as a biofilm associated shield. In line with the above experiments, mechanical disruption of surface attached biofilms was reported to restore the oxidative burst activity in PMNs [16], [17].

Disruption of aggregates is likely to lead to disruption of this shield, which allows PMNs to develop oxidative burst [9], [22].

**Figure 7:**
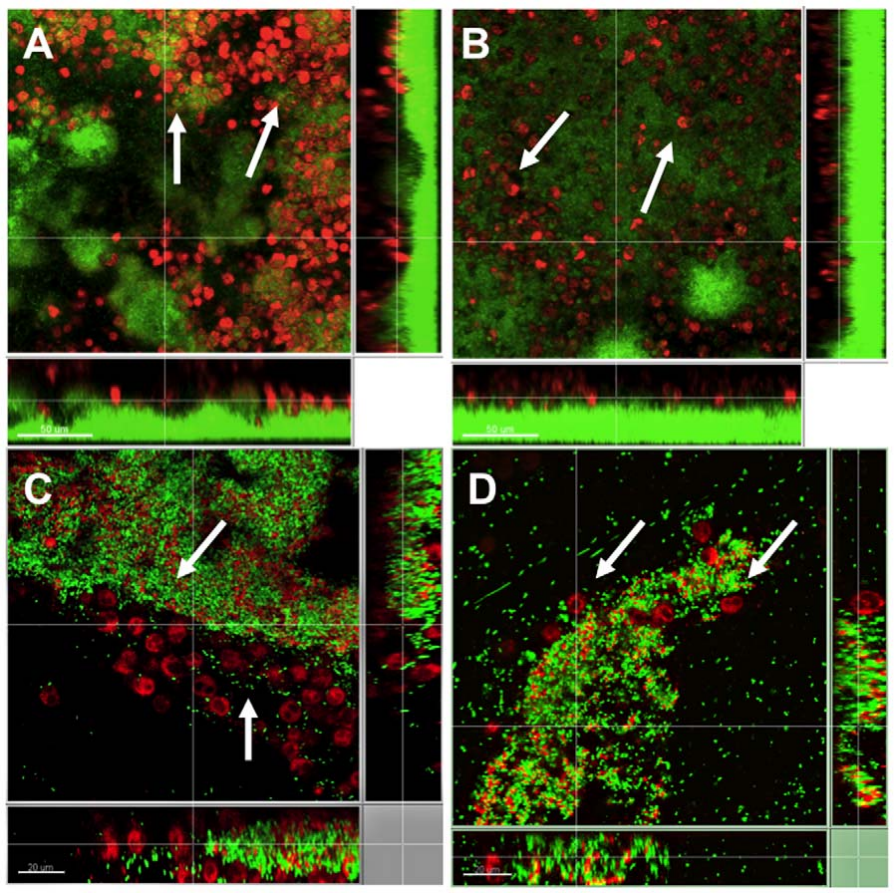
Tolerance towards PMNs. Flow-cell biofilms (A+B) were grown for 72-h before addition of PMNs and the aggregates (C+D) were grown for 48-h before the addition of PMNs. The images shows that aggregates are not phagocytosed or penetrated by PMNs. For visualization a GFP-tagged PAO1 strain (green) was used and SYTO62 was used to stain the PMNs (red). Arrows point at paralyzed PMNs. Length of size bars: 20 µm.

As this study has shown striking similarities between aggregates obtained from stationary cultures and otherwise surface attached biofilms, we speculated that the aggregates may also have the capability to resist attack from phagocytes. We therefore isolated PMNs from healthy volunteers and added them to both isolated aggregates and biofilms. By CLSM and time-lapse footage we found that planktonic bacteria surrounding the large aggregated cells were readily phagocytosed by the freshly isolated PMNs; however, both biofilm (Figure 7AB) and aggregates (Figure 7CD) seemed to paralyze the PMNs at the surfaces of the free-floating aggregate indicating that they actively resist phagocytosis by PMNs. A rhamnolipid shield similar to surface attached biofilms could explain this resistance.

### Epi-fluorescent detection of extracellular DNA

**Figure 8:**
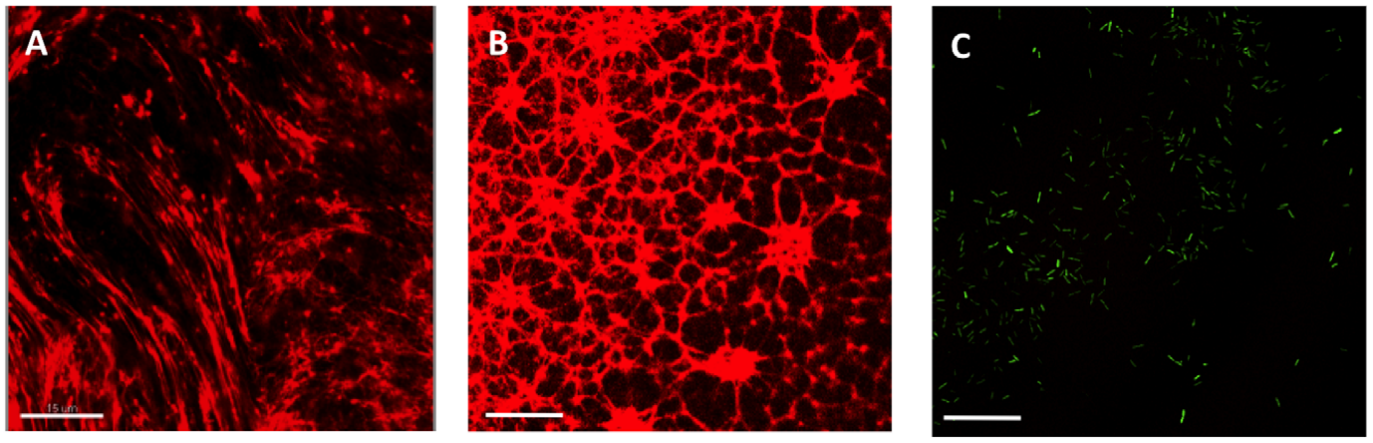
DNA content in *P. aeruginosa* cultures. DNA (PI) staining of A - Aggregate harvested from a 48-h old stationary culture stained with PI. B – 3 day old biofilm grown in flow-cell stained with PI. C – GFP-tagged planktonic cells (OD – 0.5) stained with PI. Length of size bar: 15 µm.

The fiber-like structures, which were identified using SEM, resemble the extracellular DNA previously visualized in flow-cell biofilms using PI [34]. To investigate whether our non-surface generated aggregates produced extracellular DNA, we stained harvested samples from our three settings with PI. Both the non-surface attached aggregates (Figure 8A) and the surface bound biofilm (Figure 8B) produced extracellular DNA, whereas the planktonically-growing bacteria, as expected, were not producing any extracellular DNA (Figure 8C). This indicates that the production of extracellular DNA is linked to the aggregation process or vice versa. This is in accordance with a previous publication by Allesen-Holm *et al*, which show that the production of extracellular DNA is controlled by the cell density regulatory mechanism quorum sensing. Additionally, they showed that extracellular DNA stabilizes the three-dimensional structure of flow-cell biofilms [34].

**Figure 9:**
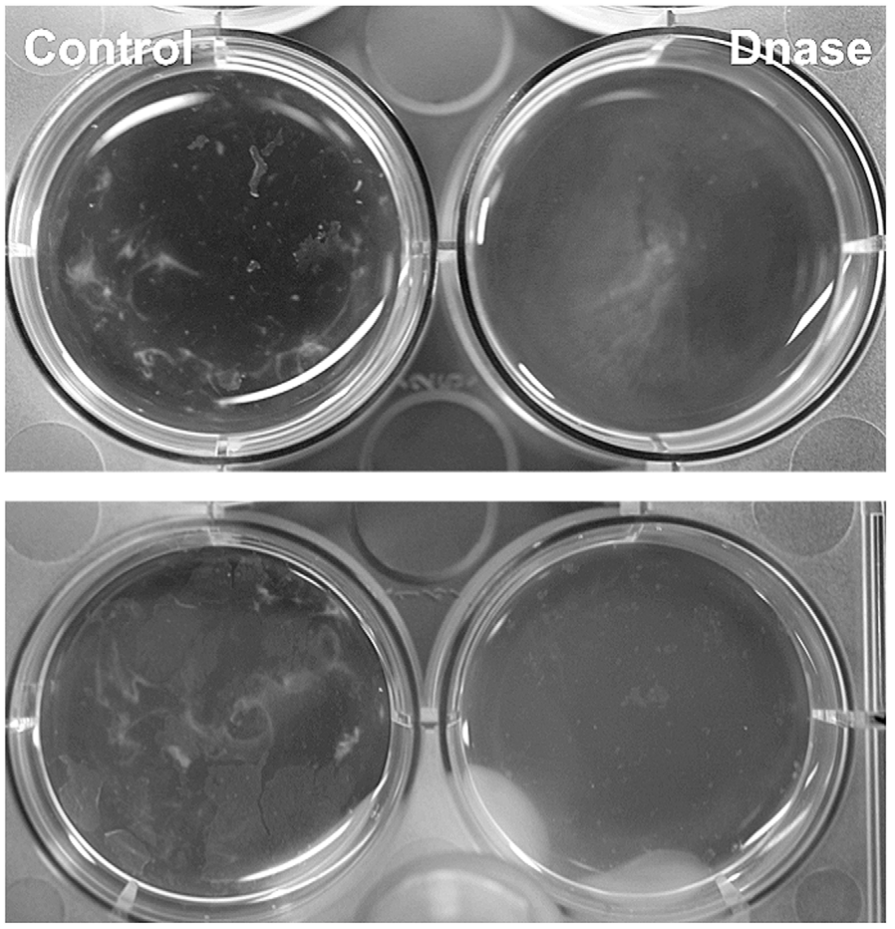
Effect of DNase on aggregate formation. Stationary cultures grown with 90 U/ml DNase I in the medium. After 48-h there was a clear difference of the visible aggregation in the culture. The control was left untreated. The top and bottom panels represent two independent experiments.

Recently, studies have shown that treatment of biofilms with DNase obstruct surface attached biofilms at early stages. Older biofilms seemed to be independent of the structural requirement of extracellular DNA. Interestingly, the same study showed that biofilm formation was prevented by the presence of DNase in the growth medium [35]. This inspired us to grow the aggregates in the presence of DNase. The presence of DNase during conditions of growth clearly reduced the macroscopic and visible aggregates in the culture (Figure 9) without affecting growth (data not shown). However, the DNase did not completely remove the fiber structures seen with SEM indicating that the fiber-like structure is probably not solely DNA (Figure 9). These data suggest a similar role for DNA in the extracellular matrix of aggregates. In fact, these data highlight that an extracellular matrix is an important feature of aggregates.

**Figure 10:**
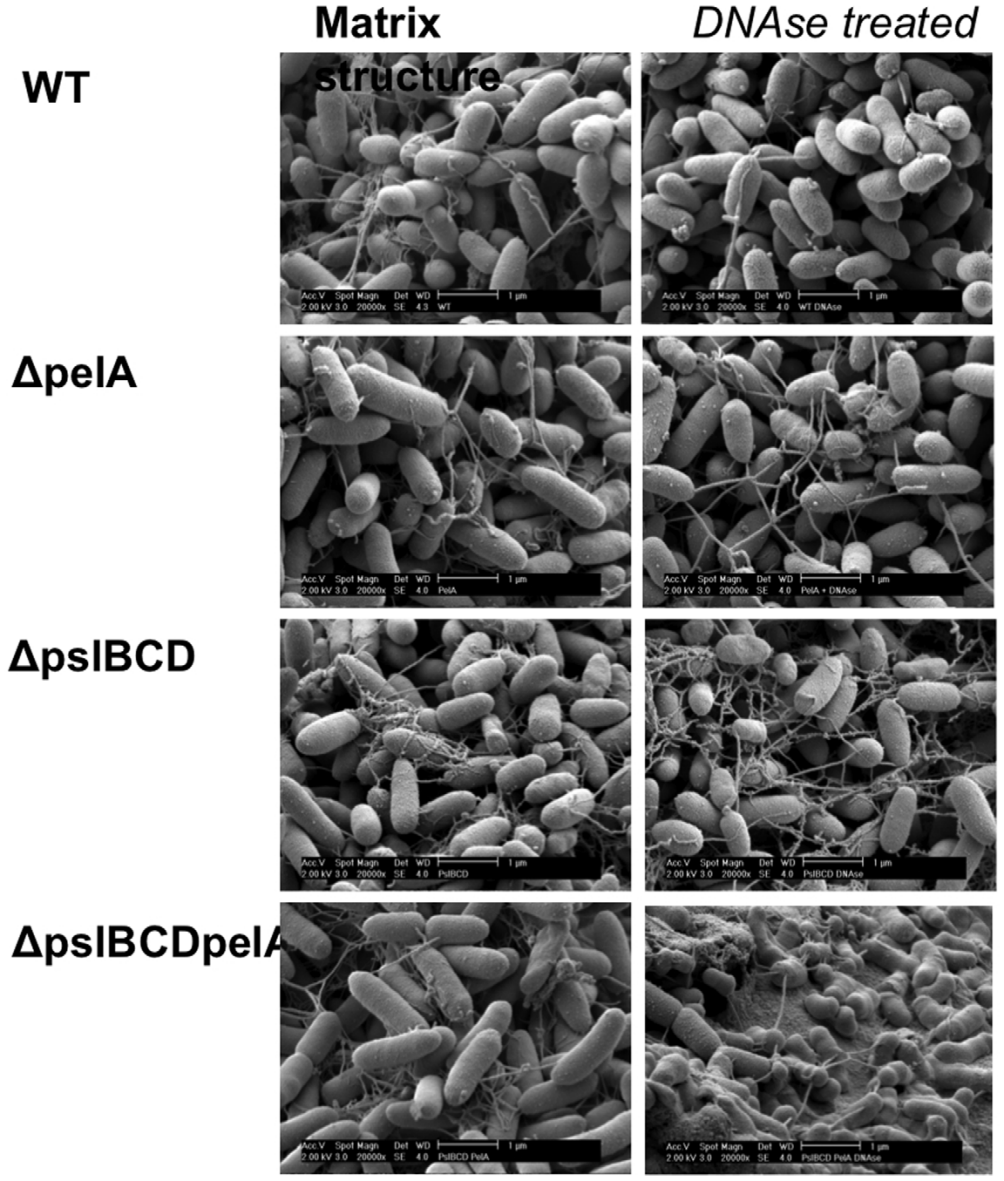
Matrix production by mutants. We tested whether if mutants not able to produce the two distinct extracellular polysaccharides (Pel and Psl) could form intercellular fibers and if these fibers could be removed by DNAse treatment. WT - wild type PAO1, ΔpelA – PAO1 not able to produce Pel polysaccarides, ΔpslBCD - PAO1 not able to produce Psl polysaccarides, ΔpslBCDpelA - PAO1 not able to produce both Psl and Pel polysaccarides.

Since polysaccharides and proteins have been shown to be major components of the matrix [36], [37], [38], [39] we speculated that the structures of the fibers could be a mixture of polysaccharides and DNA. We hypothesized that DNA may serve as a scaffold on which the other components can attach and form thick fibers. Especially, the Pel and Psl polysaccharides have been found to be important constituents of the extracellular matrix found in biofilms [36], [37], [40], [41]. The Pel polysaccharide is a glucose-rich polymer that primarily plays a role after surface attachment [37], [42]. Psl has been described as a repeating pentasaccharide containing D-mannose, D-glucose and L-rhamnose important for attachment to abiotic and biotic surfaces [36], [43]. Psl also has an important role maintaining biofilm structure post-attachment [44]. Since these polymers seem to play an important role in the matrix, we speculated that the observed fibers may indeed contain polysaccharides. We therefore utilized *psl* and *pel* mutants, and grew them in static cultures to see if they formed aggregates as the wild type and whether if they produced the fibers. In line with their biofilm forming abilities, we found that the mutants were impaired in gross scale formation of aggregates. However, the very few aggregates formed were also interconnected by some fiber structures. Consequently, solely these polysaccharides or DNA does not form the fibers. Thus it seems that the fiber could be a joint structure produced from a variety of different matrix components (Figure 10).

**Figure 11:**
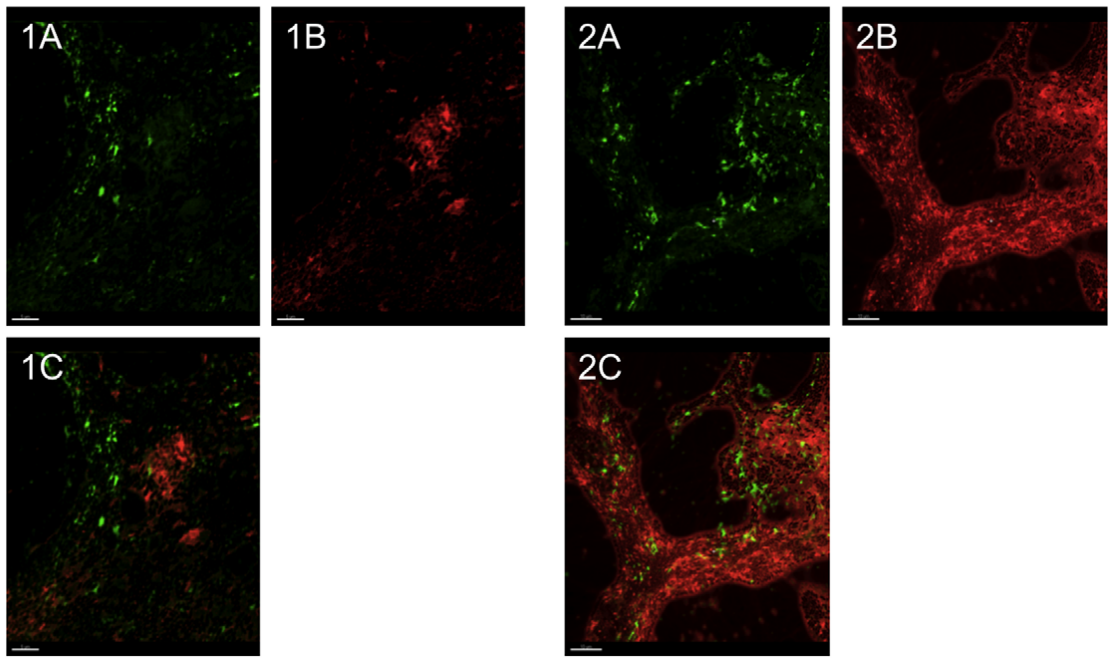
Localization of lections and DNA in aggregates. HHA-FITC and PI staining of aggregate harvested from a 48-h old stationary culture. Figure 1 and 2 represent two independent experiments with A, B and C representing HHA-FITC, PI and HHA-FITC+PI. To test whether if the fibers are made from more than one polymer, we co-stained the aggregates with PI (red) and a mannose-specific lectin stain (HHA-FITC) to visualize DNA and any present Psl polymers. Length of size bar; Figure 1: 5 µm; Figure 2: 10 µm.

To test whether if the fibers are made from more than one polymer, we co-stained the aggregates with PI and a mannose-specific lectin stain (HHA-FITC) to visualize DNA and any present Psl polymers (Figure 11). We found that Psl (green) co-localized with the extracellular DNA (red). The majority of the mannose-rich polymers were found in near presence of the red stained DNA, but we were not able to visualize any joint-polymers consisting of both DNA and Psl. This could explain why it was not possible to remove all fibers with DNAse treatment since the remaining fibers could be mannose-rich polysaccharides such as Psl.

## Discussion

Medically relevant bacterial biofilms are generally defined as a multi-cellular surface associated community embedded in a self-made exopolymeric matrix. This definition has led to confusion in the field as to whether suspended cellular aggregates represented the biofilm state of growth. This distinction has been particularly important when considering the importance of biofilms in certain chronic infections. However, two clearly identified phenotypes associated with surface attached biofilms are the aggregation of cells and a significant increase in the capacity to cope with antibiotic treatments. Bacterial biofilms seem to be the important cause of chronic infections in patients. Our previous findings have shown that bacteria found at the focus of chronic infections tend to aggregate, but are not necessarily attached to a surface. In this study we have shown that similar *in vitro* aggregates are very capable to resist high doses of antimicrobials as well as phagocytic cells.

On the basis of this we propose to include non-attached aggregates in the biofilm definition, as structures capable of resisting the antimicrobial activities of antibiotics and host immune cells.

The important question is what drives *P. aeruginosa* to produce or form an obviously protective aggregate or biofilm? This question remains to be resolved but we speculate that biofilm formation (even in the absence of a surface) may be the default mode of growth, which offer sufficient shielding to environmental insult.

These studies emphasize the importance of the *in vitro* conditions in which an experimental biofilm is studied. Even though a given bacterial isolate fails to form a biofilm at the vacant surface of a flow-cell or in the bottom of a microtiter dish, it may still form “floating” or detached aggregates fully capable to persist in hostile environments. *In vitro* surface-attachment is by many seen as a hallmark of a bacteriums pathogenic capacity. Since we have observed non-attached aggregates in chronic infections of lungs of CF patients, otitis media, soft tissue fillers and non-healing wounds, we put forward that aggregated cells exhibit the required survival capability in the hostile human environment and therefore can be considered to fully represent the biofilm growth phenotype.

We here show an inherent antibiotic tolerance to antibiotics and phagocytes enabled by bacterial aggregation that we believe is reversible and not irreversible like antibiotic resistance. As with the surface attached biofilms, it seems that the tolerance towards antibiotics relies on age and thus physiological states within the aggregate, but also properties of the matrix it self (eg slow penetration and low oxygen). In this study we have shown that these aggregated cells can be eradicated by a combination of antibiotics and simple mechanical disruption. We suggest that if disruption procedures, no matter whether they are physical or chemical in nature can be administered *in vivo* together with high concentrations of local administered antibiotics, we may be able to more efficiently eradicate chronic infections.

## Materials and Methods

### Bacterial Strains

The *P. aeruginosa* PAO1 wild-type used for the experiments was obtained from the Pseudomonas Genetic Stock Center (http://www.pseudomonas.med.ecu.edu, strain PAO0001). Construction of Δ*pel* and Δ*psl* strains were In the study we also used polysaccharide knockout mutants Δ*pelA*, Δ*pslBCD* and Δ*pslBCDpelA*. All mutants were made from *P. aeruginosa* PAO1. The construction of the Δ*pel* and Δ*psl* strains was performed as previously described by [45], [46].

### Growth of bacteria

All bacteria were grown in ABtrace minimal medium containing 0.3 mM glucose for continuous cultures and 0.5% glucose for static cultures as well as batch cultures, as described previously by [9].

Planktonic cultures were grown in shake flasks at 37°C. To obtain a pure planktonic culture an overnight culture was diluted in fresh media to OD_600_ 0.1, re-grown to OD_600_ 0.5, diluted in fresh media to OD_600_ 0.1, and again re-grown to OD_600_ 0.5.

Continuous biofilms were cultivated in once through flow chambers, perfused with sterile media, as described previously [9]. Antibiotic tolerances were determined as described in [9]. Staining of extracellular DNA was performed using PI as described elsewhere [34].

Static cultures were cultivated in 10 ml of sterile media by inoculating each well of 6 well tissue culture test plate (Techno Plastic Products, Schwitzerland) with 100 µl from an overnight culture of bacteria. Spontaneous aggregation was allowed to develop for 48 h at 37°C. Formed macroscopic aggregates from wells in triplicates were isolated by removing growth media (including non aggregated bacteria) and either transferred to new clean wells with fresh medium for controls, transferred to clean wells with fresh medium containing antibiotics, harvested and whirly mixed (for indicated time) then moved to clean wells with fresh medium containing antibiotics. The aspirated bacteria (non aggregated cells) were included as a control. All the well cultures were then grown 24 h at 37°C. On day 3 after 24 h antibiotic serial diluted and plated for treatment or control, all aggregates were collected separately and washed 3 times with sterile saline. Finally, after this all the aggregates were disrupted using ultra sound and whirly mixed, followed by determination of CFUs.

### PMN exposure and phagocytic experiments

Fresh PMNs from human volunteers were isolated as previously described by [47]. One million PMNs resuspended in 10 µl of Krebs-Ringer buffer supplemented with 10 mM Glucose were added to isolated aggregates from 48-h stationary cultures of *P. aeruginosa*. The ratio of PMNs was estimated by light microscopy to be approximately 1 PMN per 1000 bacterial cells.

### Lectin and DNA staining

FITC Conjugated Hippeastrum hybrid Lectin (Amaryllis) HHA (detects either 1,3- or 1,6-linked mannosyl units in polysaccharides) were used at a final concentration of 100–200 µg/ml as previously described by [43]. For lectin and PI double staining, biofilms were first stained by PI (30 µM final concentration) for 15 min. After three washes, PI stained biofilms were stained by FITC-labeled lectins for 2 h in the dark and observed by Confocal laser Scanning Microscopy (CLSM).

### Confocal Laser Scanning Microscopy (CLSM)

Visual biofilm development, antibiotic tolerance, phagocyte tolerance and presence of extracellular DNA were examined by CLSM using a Leica SP5 (Leica Microsystems GmbH, Wetzlar, Germany) equipped with an argon laser for excitation of the fluorophores. Simulated fluorescence projections and vertical cross-sections through the biofilms were generated by using the IMARIS software package (Bitplane AG, Schwitzerland). Images were further processed for display by using the PhotoShop software (Adobe, USA).

### Scanning Electron Microscopy (SEM)

The aggregates from all culture setups and their extracellular structures were imaged by SEM as previously described in [48]. Briefly, bacteria were harvested and fixed in 2% glutaraldehyde, post-fixed in 1% OsO_4_, critical point dried using CO_2_ and sputter coated with gold according to standard procedures. Specimens for SEM were investigated with a Philips XL Feg30 SEM microscope operated at 2–5 kV accelerating tension.

### rRNA quantification

#### Production of spike-in

To determine the loss of DNA and RNA during the DNA/RNA purification process, nuclease treatment and cDNA generation DNA and RNA were analysed with spike-ins. The *E. coli* 16 s rDNA was used as the DNA spike-in and produced by *in vitro* transcriptional PCR (IVT PCR). An overnight culture of wild type *E. coli* (MG1655) was lysed and DNA was purified with the Qiagen DNaesy Tissue Kit following the manufacturer's instructions. IVT PCR was run on a mixture of 2 µl template DNA, 1 µl 25 mM MgCl2, 4 µl 2.5 mM dNTPs mix, 1 µl 20 µM forward primer (5′-GCT ACA ATG GCG CAT ACA AA-3′), 1 µl 20 µM reverse primer (5′-TTC ATG GAG TCG AGT TGC AG-3′) (Lee *et al.*, 2008), 0.5 µl tag-polymerase, 5 µl PCR-buffer 10×, 35.5 µl MiliQ H2O to a final volume of 50 µl.

The PCR program was as follows: 95°C for 2 min, 30× (95°C for 30 sec, 55°C for 30 sec, 72°C for 1 min), 72°C for 5 min, 4°C ∞ min. The PCR-product was purified with PCR Clean Kit QiAprep according to the manufacturer's instructions. The purified product was run on a 1% agarose gel with 0.001% Etylium bromide to confirm that there was only one band of 101 bp following the PCR reaction.

Promega Luciferase Control RNA 1 mg ml-1 was used as the RNA control spike-in. The control RNA consisted of 1807 bp, which was confirmed on a 1% agarose gel with 0.001% Etylium bromide.

### DNA and RNA purification

Samples were thawed on ice and subsequently homogenized by vortexing. The 1.8 ml samples were transferred to 2 ml Eppendorf tubes and were centrifuged at 16000 g for 5 min. The supernatant was removed. The pellet was re-suspended in 0.5 ml CTAB 10% extraction buffer and transferred to a Mobio Glass Bead Tube (0.5 mm), together with 0.5 ml of Fluka, phenol-chloroform-isoamyl alcohol (25∶24∶1). Bead tubes were beaded on MoBio Bead beading Vortex Adapter for 2×60 sec separated by 60 sec on ice. At this point 5 µl of 1 ng µl-1 Promega Luciferase Control RNA (lucI RNA) and 5 µl of 10-1 dilution of *E. coli* 16 s PCR product were added as spike-in controls. The tubes were then centrifuged at 16000 g for 5 min at 4°C. The upper portion of the supernatant (about 425 µl) was transferred to a new 2 ml Eppendorf tube. Phenols were removed by adding 425 µl of Fluka, chloroform-isoamyl alcohol (24∶1) and mixed gently by pipetting. These were then centrifuged at 16000 g for 5 min at 4°C. The upper phase (approximately 400 µl) was transferred to a new Eppendorf tube without touching the interphase. 800 µl 30% PEG and 1 µl glycogen were added to the new tube, followed by gentle mixing by pipetting. The tube was put on ice in a Styrofoam box for 2 hours. Subsequently, the tube was centrifuged for 30 min at 16000 g and 4°C. The supernatant was removed carefully without disturbing the very small pellet. The pellet was re-suspended in 50 µl nuclease-free water.

### On column purification of RNA

The 50 µl sample was further purified using the Qiagen RNeasy Mini Kit. The 50 µl were added to 350 µl RLT buffer and 250 µl 96% ethanol. The mixture was mixed by pipetting and transferred to the septum in the RNeasy Mini spin column and was centrifuged at 8000 g for 15 sec. Flow-through was removed and 500 µl RPE buffer containing the column were again centrifuged at 8000 g for 15 sec. Flow-through was removed and 500 µl RPE buffer with the column were again centrifuged at 8000 g for 2 min. The column was then moved to a new collection tube and centrifuged at full speed for 1 minute to dry the septum. The column was placed in a DNase/RNase-free 1.5 ml Eppendorf tube and 55 µl nuclease-free water was added to the membrane and centrifuged for 1 min at full speed. This produced about 52 µl DNA/RNA with throughput. The purified product was divided into two equal parts (25 µl of each portion).

### Removal of DNA and RNA, respectively

RNA was removed from one portion and DNA was removed from the other. DNA was removed by adding 7.5 µl DNase I (Ambion), 5 µl 10× reaction buffer (Ambion) and 12.5 µl RNase-free water. This mixture was incubated at 37°C for one hour. Subsequently, 7.5 µl DNase stop solution (Ambion) was added. After 5 min at room temp., 42.5 µl RNase-free water was added to obtain a total volume of 100 µl. RNA was removed by adding 37.5 µl 1 N NaOH and incubating for 30 min at 65°C. Subsequently, the NaOH was neutralized with 37.5 µl 1 N HCl. Samples were run on a 1% agarose gel with 0.001% Etylium bromide to ensure that all rRNA had been removed from the DNA samples. Samples that contained RNA were frozen at −80°C. Samples of DNA were frozen at −20°C.

### cDNA Synthesis

The purified DNA and RNA samples, respectively, were thawed on ice. RNA samples were then turned into cDNA by Reverse Transcription (RT) using the Applied Biosystems (AB) High Capacity RNA-to-cDNA Master Mix. The reaction was a mixture of 4 µl AB High Capacity RNA-to-cDNA Master Mix and a sufficient volume of sample (max 16 µl) so that the content of RNA was as close to 1 µg of each reaction mix as possible. (The volume should be taken into account in the data analysis). Reaction mix was adjusted with RNase/nucleotide-free water to a final volume of 20 µl. The mixture was made up in AxyGen 0.2 ml PCR-strips tubes. The PCR was performed in an AB 2720 Thermal Cycle with the following program: 25°C for 5 min, 42°C for 30 min, 85°C for 5 min, 4°C for ∞ min. Samples were placed in a −20°C freezer immediately after the completion of the program.

### RT-PCR run

cDNA from RT was diluted 100 times prior to RT-PCR. DNA and cDNA samples were thawed on ice. RT-PCR reaction was carried out in an AB MicroAmp 96-well 0.1 ml reaction plate. Samples were tested for the amount of *P. aeruginosa* 16 s rDNA, 16 s rRNA and the spike-in lucl RNA and *E.coli* 16 s DNA. The primers used for *P. aeruginosa* 16 s rDNA and rRNA were: Forward 5′-CAA AAC TAC TGA GCT AGA GTA CG-3′ and reverse 5′-TAA GAT CTC AAG GAT CCC AAC GGC T-3′ (Matsuda *et al.*, 2007). For spike-in lucI RNA: Forward 5′-GTG TTG GGC GCG TTA TTT ATC-3′ and reverse 5′-ACT GTT GAG CAA TTC ACG TTC-3′. For spike-in *E.coli* 16 s rDNA: Forward 5′-GCT ACA ATG GCG CAT ACA AA-3′ and reverse 5′-TTC ATG GAG TCG AGT TGC AG-3′ (Lee *et al.*, 2008). All primers were tested and verified with NCBI BLAST and NCBI Primer BLAST. There was no overlapping of the primers and eukaryotic genomes. No convergence was found between *P. aeruginosa* 16 s primer and spike-in or vice versa. The RT-PCR reaction mix consisted of 10 µl AB SYBR Green PCR Master Mix, 0.2 µl Forward primer, 0.2 µl Reverse primer, 7.6 µl nuclease-free water from Qiagen and 2 µl sample to a final volume of 20 µl in each well. The plate was sealed with MicroAmp foil. RT-PCR reaction was run on AB StepOnePlus Real-Time PCR System with the following reaction program: 95°C for 15 sec then 40× (95°C for 15 sec, 60°C for 1 min), 95°C for 15 sec, 60°C for 1 min, Melting curve with a 0.3°C stepwise increase in temperature to 95°C. A stepwise decrease in target sequence concentrations of 10-1 was used for quantification standard curves. All reactions were performed in duplicate.

### Data analysis and spike-loss

Results obtained by RT-PCR were processed in Excel. The loss of DNA and RNA during purification and further processing was taken into account as the measured loss of the DNA and RNA spike-ins.

### Statistics

All statistical analyses (except Cuziack's test for trend) were performed with GraphPad Prism version 4.00 for macintosh, GraphPad Software, San Diego California USA, www.graphpad.com. Cuziack's test for trend were performed manually.

Kolmogorov-Smirnov tests for normality were applied on the antibiotic treatment experiments.

Figure 4: The test for normality did not confirm Gaussian distribution in all data sets – therefore a nonparametric Mann-Whitney test were chosen to compare the medians of the different treatment regimens.

Figure 5: The test for normality did not confirm Gaussian distribution in all data sets. The Kruskal-Wallis test was chosen to compare the effect of whirly mix and subsequent treatment with tobramycin. To test if longer periods of whirly mix yielded enhanced killing a Cuziack's test for trend was performed on the whirly mixed groups. Further, the Kruskal-Wallis test was followed by a post test (Dunn's) to determine the minimum time of whirly mix before a significant reduction in viable bacteria was observed.

### Ethics statement

This research did not involve human or animal participants and therefore there is no requirement for an ethics statement.
